# Adaptive work in the primary health care response to domestic violence in occupied Palestinian territory: a qualitative evaluation using Extended Normalisation Process Theory

**DOI:** 10.1186/s12875-020-01338-z

**Published:** 2021-01-02

**Authors:** Loraine J. Bacchus, Abdulsalam Alkaiyat, Amira Shaheen, Ahmed S. Alkhayyat, Heba Owda, Rana Halaseh, Ibrahim Jeries, Gene Feder, Rihab Sandouka, Manuela Colombini

**Affiliations:** 1 London School of Hygiene & Tropical Medicine, Department of Global Health and Development, Faculty of Public Health & Policy, 15-17 Tavistock Place, London, WC1H 9SH UK; 2 Public Health Department, An-Najah National University, Faculty of Medicine and Health Sciences, P.O. Box 7, Nablus, West Bank Palestine; 3 University of Bristol, Population Health Sciences, Canynge Hall, 39 Whatley Road, Bristol, BS8 2PS UK; 4 Juzoor for Health and Social Development, Palestine P.O. Box 17333, Jerusalem,

## Abstract

**Background:**

A health system response to domestic violence against women is a global priority. However, little is known about how these health system interventions work in low-and-middle-income countries where there are greater structural barriers. Studies have failed to explore how context-intervention interactions affect implementation processes. Healthcare Responding to Violence and Abuse aimed to strengthen the primary healthcare response to domestic violence in occupied Palestinian territory. We explored the adaptive work that participants engaged in to negotiate contextual constraints.

**Methods:**

The qualitative study involved 18 participants at two primary health care clinics and included five women patients, seven primary health care providers, two clinic case managers, two Ministry of Health based gender-based violence focal points and two domestic violence trainers. Semi-structured interviews were used to elicit participants’ experiences of engaging with HERA, challenges encountered and how these were negotiated. Data were analysed using thematic analysis drawing on Extended Normalisation Process Theory. We collected clinic data on identification and referral of domestic violence cases and training attendance.

**Results:**

HERA interacted with political, sociocultural and economic aspects of the context in Palestine. The political occupation restricted women’s movement and access to support services, whilst the concomitant lack of police protection left providers and women feeling exposed to acts of family retaliation. This was interwoven with cultural values that influenced participants’ choices as they negotiated normative structures that reinforce violence against women. Participants engaged in adaptive work to negotiate these challenges and ensure that implementation was safe and workable. Narratives highlight the use of subterfuge, hidden forms of agency, governing behaviours, controls over knowledge and discretionary actions. The care pathway did not work as anticipated, as most women chose not to access external support. An emergent feature of the intervention was the ability of the clinic case managers to improvise their role.

**Conclusions:**

Flexible use of ENPT helped to surface practices the providers and women patients engaged in to make HERA workable. The findings have implications for the transferability of evidenced based interventions on health system response to violence against women in diverse contexts, and how HERA can be sustained in the long-term.

## Background

Violence by an intimate partner (IPV) affects one in three women globally and is associated with adverse health consequences [1]. A health system response is a global priority [2] especially in low-and-middle-income countries where the prevalence is high [1]. The Palestinian Authority reports a 29% life time prevalence of any form of IPV (psychological, physical, sexual, social or economic) experienced by women in the occupied Palestinian Territory (oPt), which was the setting for our case study [3].

Much of the evidence on health sector interventions that address IPV is skewed toward high-income countries [2]. Little is known about whether or how they work in low-and-middle-income countries where there are general deficiencies in the funding, organisation and delivery of health care [4] and a scarcity of specialised services to which women experiencing violence can be referred [2].

A health system approach has been advocated by systems researchers, which attends to the influence of broad contextual factors such as resources, infrastructure, leadership and governance, multi-sectoral coordination, monitoring and health workforce issues [2, 5]. This is important for understanding how health system interventions that integrate a domestic violence response are made workable and sustained in diverse contexts and for scaling up interventions that are proven to be effective [6]. However, few evaluations of these interventions draw on theories that permit such exploration [7–9].

### Defining context and complexity

When thinking about IPV interventions in healthcare, the context is broader than the health system because it contains features of political, economic and socio-cultural contexts through which the implementation process will proceed [10]. Exogenous factors in the outer setting such as economic crisis, changes of government, law and policy and epidemics can cause turbulence to the system (the inner setting) [11]. Whilst the boundary between the inner and outer setting is not always clear the interaction between them is often dynamic and precarious [10]. It has been argued that the meaning of *complexity* in complex interventions has not been problematized enough, resulting in an over-simplistic focus on articulating the active ingredients or mechanisms underpinning interventions and measuring them as discrete elements [12]. A significant aspect of complexity lies not in the intervention itself, but in that context into which the intervention is introduced, how it interacts with the context and the adaptations that emerge from this [13]. May et al. [6] describe context as “an unstable, unfolding process*”* as opposed to a fixed organisational structure in which the intervention takes place. A critical review of representations of context within research on health interventions found that few studies robustly described the synergy between intervention and context and tended to treat context as a physical setting or something to be controlled for [14].

### Context in Palestine

**Fig. 1:**
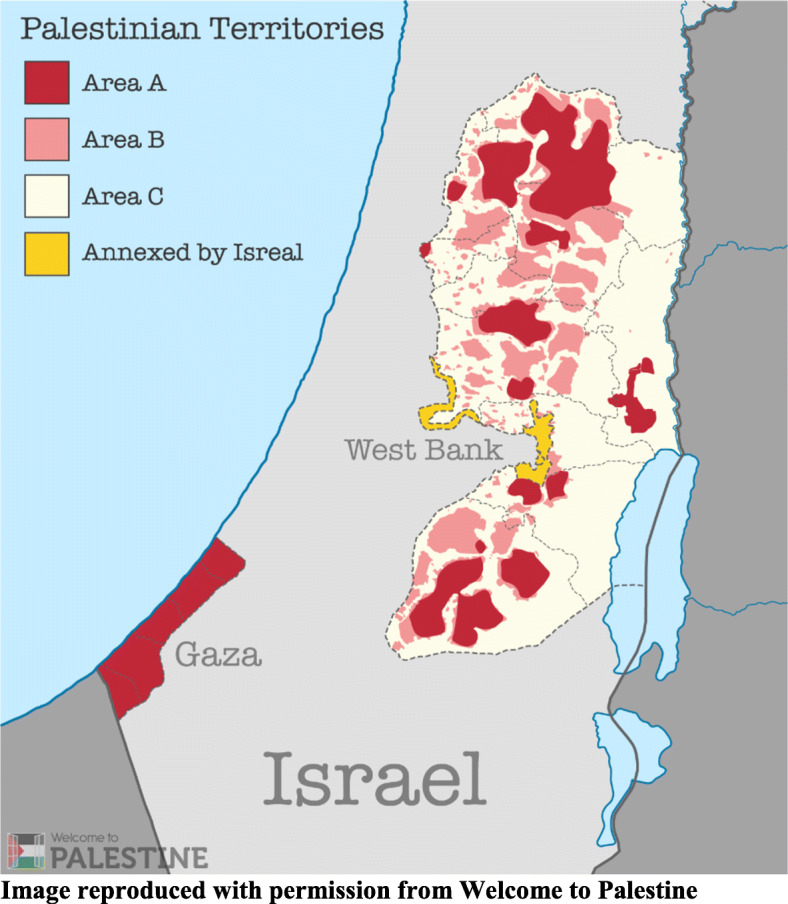
Palestinian Territories, Image reproduced with permission from Welcome to Palestine

The case study described in this paper is an intervention which aimed to strengthen the primary healthcare response to domestic violence in occupied Palestinian territory (oPt), defined here as any form of violence or abuse by a “household member”, including a current or former husband or family [3]. It was anticipated that the intervention would interact with political, sociocultural and economic aspects of the context, creating a degree of uncertainty during its implementation. Palestinians have been living under Israeli military occupation for over 50 years. Under the Oslo II Accord the West Bank, the setting for this study, is divided in to Areas A, B and C (Fig. 1). The Palestinian Authority assumes civil authority (i.e. infrastructure, planning, building) and security control in Area A whilst sharing only security responsibility with Israel in Area B. Area C, which represents more than 60% of the West Bank and contains the most valuable natural resources remains under full Israeli security and civil control. The concomitant legislative and physical division of oPt restricts the free movement of Palestinians, as well as their access to basic services such as health and education, and separates communities from their land, places of work and social networks [15]. During the period of the study, there was a reported increase in violent incidents between Palestinians and Israeli forces in the West Bank [16].

Feminist scholarship is also relevant to understanding the context in which the intervention took place. Women and girls’ vulnerability to violence is rooted in the greater power and value that societies confer upon men and boys, regarding access to material, symbolic and relational resources compared with women and girls [17]. Arab culture maintains a strong hierarchy where older aged people and especially males are held in high esteem in terms of household decision making. The traditional and still widespread family model sees men as the providers and a source of protection, and women as primary care givers and nurturers. Childcare, care of the elderly and those with disabilities within the household is usually born by family members, with females bearing most of the responsibility. In Palestine the median age of marriage among females is 23 years and 24 years among males. Polygamy is not uncommon and having many children is perceived as a form of social security and protection as many Palestinian families have lost members as a result of the ongoing political violence [18]. Divorced women experience economic and social disadvantage and the prospects of re-marriage are often poor. Consequently, women may endure years of marital conflict in order to avoid the stigma associated with divorce and losing their children. Receiving social, economic and familial support may be tacitly conditional on adhering to traditional norms [19].

The prolonged political conflict in oPt, can place stress on gendered relations as the traditional role of the man as protector and provider for the family is diminished as a result of poverty, unemployment and humiliation. This can manifest as increasing levels of violence within the family towards those with less power such as women and children [20, 21]. The deterioration of the economic and security situation caused by the occupation, alongside laws, tribal systems and institutions that reinforce patriarchal ideology, further restricts women and curtails their ability to seek solutions. Studies from the oPt document a link between occupation-related adversity and experiences or perpetration of IPV [20–23].

## Methods

### Aims of the study

Our study aimed to explore the broader structural and contextual factors that emerged as troubling or supporting the implementation process in an intervention to address domestic violence in primary health care settings in oPt. Furthermore, we wanted to understand how these were negotiated by primary health care providers and women service users.

### Implementation theory

**Fig. 2:**
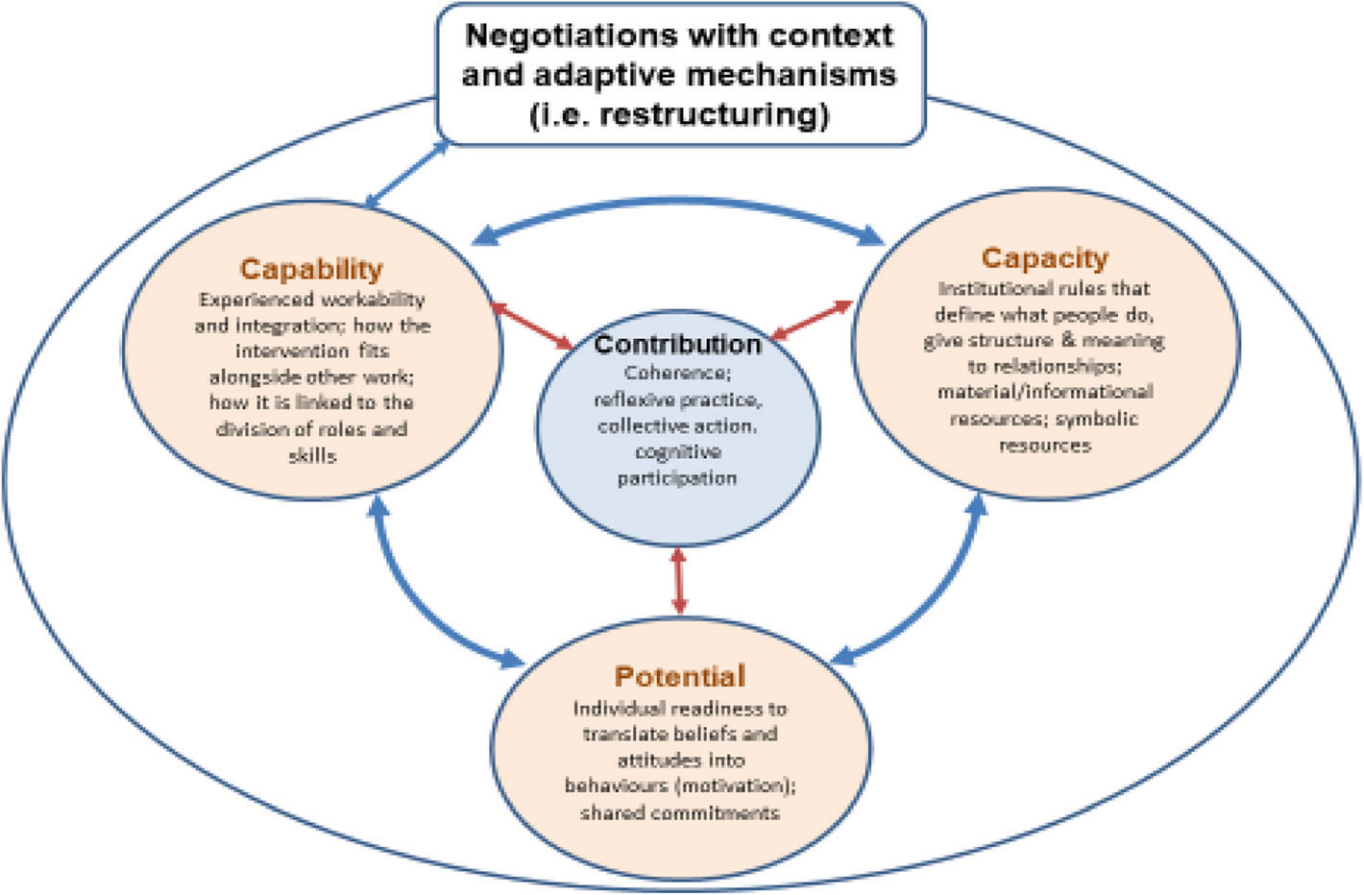
ENPT constructs adapted from May et al. [11]

The choice of implementation theory, Extended Normalisation Process Theory (ENPT), was guided by the need to engage with the uncertainty of the intervention mechanisms as it interacted with the broader context. We chose ENPT over an earlier iteration of the theory (Normalisation Process Theory - NPT) because of its attention to the dynamic role of implementation contexts in the mobilisation and negotiation of implementation processes [24]. ENPT draws on ideas about complex adaptive systems (CAS) and unforeseen emergence. Systems are described as adaptive because they have the capacity to change and to self-organise (transform) when they encounter turbulence and chaos from the environment in which they are embedded. Implementation processes are emergent, in that they unfold over time and are shaped by diverse factors [6, 11]. ENPT describes two adaptive mechanisms that participants within complex interventions use to negotiate context. *Normative restructuring* in a CAS refers to modifications to the conventions, rules and resources that participants experience as providing the scaffolding for everyday work and interactions. *Relational restructuring* refers to changes in the structure and conduct of interpersonal interactions and group processes that make collective action possible. Normative and relational restructuring evolve over time and may accelerate or decelerate. However, intervention failure may occur when participants are unable to perform the degree of restructuring that is necessary to make an intervention workable [6]. As participants enact their contributions to an implementation process they adjust their behaviour and responses to each other. In ENPT, these emergent expressions of agency are referred to as users’ *capability* (i.e. experienced workability and integration of the intervention in context). Capability is linked to the other ENPT constructs of *capacity* and *potential*. The *capacity* of the context to accommodate implementation processes depends on the social-structural resources that are made available to participants. This includes institutionally sanctioned rules that give structure to meanings and relationships; social roles assumed within a social system that define what people do; symbolic and actual material resources; and informational resources. *Potential* is concerned with individual readiness to translate beliefs and attitudes into behaviours that are congruent (or not) with system norms and roles (i.e. they shape individual and collective motivation to engage with a complex intervention) [11]. Implementation processes are not linear and the constructs of capability, capacity and potential work synergistically in a continuous feedback loop (see Fig. 2).

May [24] recommends that optimal use of ENPT should be flexible and tailored drawing on specific domains within the theory to address the research problem. Therefore, it needs to be “made at home” within the study context. With this in mind we chose not to use ENPT constructs and sub-constructs as a rigid framework in which to fit data, but as sensitising devices which helped us to understand and explain the social processes through which modified practices of thinking and working were being operationalised in the intervention. We did not draw on the construct of *contribution*, which relates to an earlier iteration of the theory known as NPT. Nor did we use ENPT language to dictate the wording of the interview guides.

The evaluation was embedded within the paradigm of critical realism [25] which aligned well with ENPT. McEvoy & Richards [26] highlight key features of critical realism and its potential for evaluation research. This branch of philosophy combines a realist ontology (theory of being) with a relativist epistemology (theory of knowledge). Critical realists seek to obtain knowledge about causative or generative mechanisms which refer to the structure, powers and relations that explain what is beneath the surface (i.e. the observable world). There is also a recognition that human behaviour is not solely determined by social structures and that people are able to respond creatively to the circumstances in which they find themselves. In ENPT May [6] describes generative mechanisms as self-organising mechanisms in complex adaptive social systems, which explain differences in implementation processes over time and between settings. Thus, they play an important role in understanding intervention fidelity. ENPT also offers a way to reflect upon the interplay between individual and collective agency and the social contexts in which these actions take place. Understanding the dynamics of human agency under conditions of constraint is central to this [6]. The critical realist approach to theory-based evaluation aims to get inside the “black box” of interventions [27] to understand what works for whom, in what circumstances and why [28].

### Description of the intervention

HERA (Healthcare Responding to Violence and Abuse) includes the following components: (i) training and reinforcement to support primary health care providers in identifying and responding to domestic violence (ii) awareness raising with women patients attending the clinics (iii) documentation of domestic violence and (iv) a care pathway for women who disclose domestic violence. Intervention development was informed by findings from the formative phase which involved structured health facility observations at the two clinics, and semi-structured interviews with primary health care providers, health managers, senior policy-makers with expertise in domestic violence programming and non-governmental organisations offering domestic violence services. The formative phase used a health systems readiness framework to identify system obstacles to successful implementation. The findings were presented at a workshop with 19 key stakeholders from various government ministries and non-governmental organisations working in the field of domestic violence. The aim of the workshop was to refine the intervention pathway and gain consensus regarding the preconditions required to support implementation and achieve the intended long-term outcomes [29]. It also helped to resolve areas of uncertainty (e.g. resource implications; role of different providers; geographical/logistic barriers for referral of survivors; and locus of further support for women). These findings are reported in a separate publication [5]. The intervention also drew on WHO recommendations for a health system response to intimate partner violence [30] and a UK evidence-based domestic violence intervention for general practice called IRIS – Identification and Referral to Improve Safety [8, 31].

**Fig. 3:**
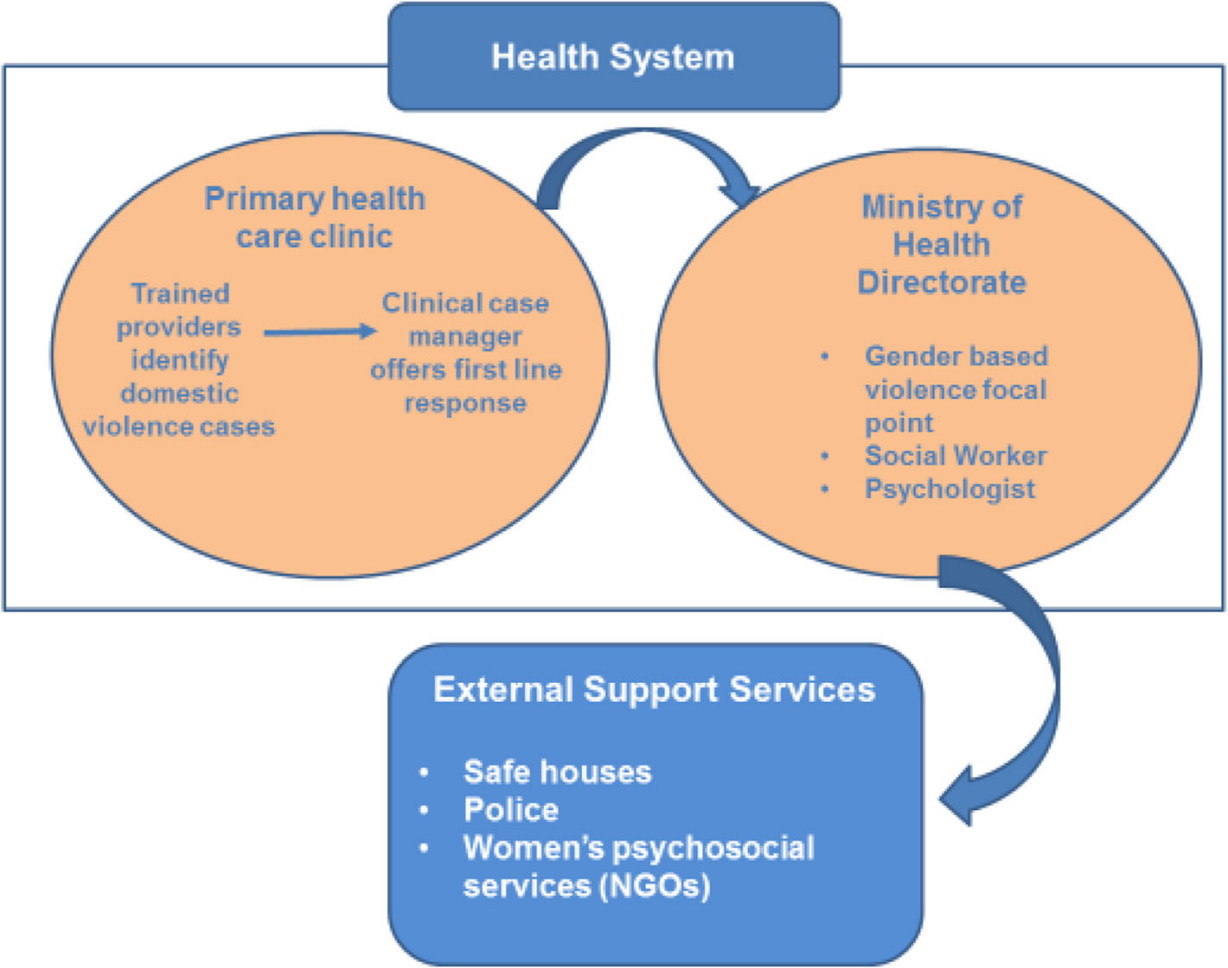
HERA care pathway

The training was developed by Juzoor for Health and Social Development with input from the research team. Juzoor is a non-governmental organisation based in Jerusalem working at the national level to improve the health and well-being of Palestinian families. An important strand of their work includes empowerment of women and protection from gender-based violence. Training sessions were delivered jointly by Juzoor and a medical doctor using didactic and experiential learning activities. Information was provided on prevalence and health impacts of domestic violence on women and children, and common presentations consistent with experiences of domestic violence. Practical sessions used group work which focused on how to sensitively ask about and respond to domestic violence including use of the care pathway and documentation. The care pathway was a core mechanism within the intervention, an organisational device to refer women experiencing domestic violence in a safe and practical way to sources of support within and outside of the health system (Fig. 3). Health care providers who identified women affected by domestic violence were trained to refer them to the clinic case manager (a nurse) who was responsible for providing a first line response, consistent with WHO guidelines on health care for women experiencing IPV [32]. This entailed empathic listening, inquiring about needs, assessing safety and offering referral to the gender based violence (GBV) focal point that was situated externally in the Health Directorate. GBV focal points coordinated additional referrals within and outside of the health system and provided advice to clinic case managers. Drawing on the experiential learning cycle [33], two training sessions for all clinic staff were delivered 3 weeks apart - to allow for reflection - between April and May 2018. Three reinforcement sessions were delivered between June and September 2018 to facilitate discussion of cases and implementation challenges. Thirteen of 17 providers in Clinic 1, and 16 of 20 providers in Clinic 2 attended two initial training sessions in April and May 2018. In total, 24 attended the first reinforcement session, 18 the second and 17 the third. The training team also conducted three domestic violence and health awareness raising sessions with 50 women patients attending the clinics. Posters on domestic violence were placed in the waiting areas of the clinics and the training team developed a brief clinic handbook, outlining the referral pathway and roles and responsibilities within it.

### Settings and ethics

The intervention was implemented in two primary health care clinics in the West Bank of oPt. Clinic 1 was based in Area C (under full Israeli civil and security control) and Clinic 2 in Area B (under Palestinian civil control, but joint Palestinian-Israeli security control). Selection was based on clinics having a reasonable infrastructure and good potential for integrating the intervention, details of which are reported elsewhere [5]. Ethical approval was obtained from An-Najah National University, University of Bristol (61603) and London School of Hygiene & Tropical Medicine (15341). Participants were asked to read an information sheet written in Arabic and written consent was obtained. The local research team received training in qualitative interviewing techniques for research on violence against women, and were encouraged to reflect on how their values and social position could impact rapport building with research participants and data quality. Four of the six researchers were medical doctors and two were Assistant Professors. Three of the researchers were female. In line with international guidance on researching domestic violence, women survivors of domestic violence were interviewed by female researchers. Most violence against women is perpetrated by men and it is recognised that women participants feel more comfortable discussing these experiences with female researchers [34]. However, given the focus of the provider interviews on implementation of HERA, it was not deemed essential to gender match, although this sometimes occurred by chance. Recent critical debates [35] highlight the complexities of building rapport in qualitative research and question the utility of matching on gender, age, ethnicity and other factors to minimise power hierarchies and enhance data quality. It is argued that matching on one characteristic does not always have the desired effect, as it may close off certain lines of questioning due to assumptions about shared world views between the participant and researcher. Furthermore, intersectionality recognises that individual identities are formed through the meeting of different social positions which can bring privilege in some areas of life, but disadvantage in others.

### Study design and data collection

The study design is a qualitative process evaluation which was undertaken between April and December 2018, consisting of interviews with a range of key stakeholders at both clinics. The design allowed a detailed exploration of the factors that shaped the translation of HERA into a real-world set of activities. Interviews were conducted in a private room within the clinics and audio-recorded with consent. We collected anonymised clinic data on identification and referral of domestic violence cases. This was collected from the clinic case registers which were used at the clinics to confidentially record disclosures of domestic violence. A member of the local research team requested anonymised clinic data on documented cases pertaining to the period of the evaluation. Provider attendance at the training sessions were collected from the domestic violence trainers. The 29 trained providers, the clinic case manager at each clinic, the domestic violence trainers and the GBV focal points from the two Directorates were invited to participate in a semi-structured interview. Clinic case managers helped to recruit women aged 18 years and over who were referred to them after disclosing domestic violence to a provider, and liaised with the research team to arrange interviews at the clinic. Non-eligible women included those who were assessed by case managers as being too psychologically distressed or affected with mental health issues which would make it difficult for them to give informed consent, although none of the women met these criteria. Twenty-one women who disclosed domestic violence and saw a case manager were approached (15 in Clinic 1 and 6 in Clinic 2). Overall, 18 semi-structured interviews were conducted with five women; seven primary health care providers, two clinic case managers, two GBV focal points and the two trainers (see Table 1). The interview guide for providers included questions related to the training and the extent to which it prepared them to deal with domestic violence cases; experiences of dealing with domestic violence cases; challenges in identifying and responding to women affected by domestic violence; documentation of domestic violence; perceptions of roles and responsibilities within the referral pathway and how the pathway worked in practice; and issues pertaining to personal safety and support for providers. The women’s interview guide included topics related to their experiences of talking to primary health providers at the clinic about domestic violence and the response they received; their feelings about referral options offered and experiences of accessing support within and outside of the health system; barriers to accessing support; changes to their situation following disclosure to a provider; issues of safety and confidentiality; and documentation of domestic violence. The interviews with providers and women patients were conducted in Arabic (AA, AS, ASA, HO, RH, IJ); the trainers were interviewed in English.

**Table 1:** Characteristics of participants

**Women**	***N*** **= 5**
**Clinic**	
1 (Area C)	3
2 (Area B)	2
**Age range**	20 to 30 years
**Length of marriage**	14 months to 11 years
**Number of children**	1 to 5
**Education**	
Secondary school	4
University	1
**Source of financial support**	
Woman’s family	4
Husband and woman’s family	1
**In paid employment**	
Yes	0
No	5
**Polygamous marriage**	
Yes	4
No	1
**Others living in husband’s home**	
Yes	3
No	2
**If yes, who**	
Husband’s family	2
Second wife of husband	1
**Professionals**	***N*** **= 13**
Clinic 1 (Area C)	5
Clinic 2 (Area B)	4
Directorate based	2
Trainers	2
**Age range**	38 to 55 years
**Sex**	
Female	9
Male	4
**Job title**	
Nurse	1
Nurse (Head nurse at clinic)	1
Midwife	1
Clinic case manager	2
GBV focal point (Directorate)	2
General practitioner (Head of clinic)	2
Family medicine doctor	1
Gynaecologist	1
Clinical co-trainer	1
NGO-trainer	1

*GBV* Gender based violence

### Data analysis

Data analysis was conducted on the English translations after they were checked for accuracy against the Arabic recordings (AA, HO, RH). Thematic analysis [36] began with members of the research team immersing themselves in the data by reading and re-reading transcripts whilst annotating with words or phrases. This was followed by in-person and remote group analysis sessions to discuss reoccurring patterns in the data and unusual views or events. These sessions were also used to achieve consensus regarding discrepant views on the emerging code frame (LJB, AA, AS, ASA, HO, RH, IJ, MC). Coding frames were developed for interviews with women and professionals and refined as additional interviews were annotated. Initial coding was mainly descriptive, drawing on topics in the interview guides which helped us to familiarise ourselves with the data and identify key challenges. In the next stage, ENPT constructs were used as sensitising devices to further interrogate the data about implementation processes, which helped to move the analysis beyond our initial descriptions of the narratives as merely reflecting a set of mutually exclusive barriers and facilitators. ENPT provided a conceptual framework within which to interpret and re-contexualise individual accounts. Whilst the initial stages of analysis used deductive and inductive approaches, the theories about structure and relations embedded within ENPT’s conceptual framework enabled abduction [37] and explanations for causal mechanisms which generated the events. ENPT helped us to think about implementation processes as being shaped by the ways that different actors worked together. We explored participants’ accounts to identify what aspects of context interacted with HERA and how they manifested, how participants made sense of them, how it impacted the way participants engaged with each other within complex social systems and how this influenced the outcomes for HERA. These were developed into a set of themes and coded extracts were reviewed to ensure each theme was coherent (LJB & MC). Nvivo 11 was used to organise and code data.

## Results

The results are divided into three sections. Themes in the following two sections (negotiating context through normative and relational restructuring) articulate the adaptive work that women and providers engaged in, how this influenced their interactions with each other, integration of HERA into existing workflows by providers and linkage to the division of roles and skills (i.e. the ENPT construct of capability). The third section (synergies with capacity and potential) demonstrates the synergistic links between *capability* and other implementation processes exemplified by the constructs of *capacity* (the socio-structural resources that are made available to intervention participants) and *potential* (individual and collective commitment).

### Normative restructuring

#### Governing behaviours (of self and others)

Interactions between health care providers and women were characterised by heightened perceptions of risk. Providers expressed anxiety about how their actions could be perceived as transgressing cultural values concerning privacy and preservation of the family, or as inciting women to leave their husbands. Potential exposure of their involvement in supporting women contributed to a fear of retaliation from the abuser or family members highlighting the challenges of changing deeply ingrained beliefs. Whilst evident in both clinics, it was a particular issue for the clinic in Area C. Deficient security arrangements under the occupation permeated and weakened the intervention, principally, on a practical level due to the absence of Palestinian police protection and in terms of curtailing the actions of providers and women.

*It hasn’t helped much [HERA training]. I do feel that my safety is vulnerable due to the lack of laws and legislation which guarantee the safety of healthcare providers. Therefore, there’s no deterrent to stop offenders from taking vengeance on the team and their families.* [HP03, GBV Focal Point, Female]

Women negotiated risks related to the potential consequences of help-seeking in a culture where strong stigmatisation was associated with speaking out about abuse. All of the women had children, were economically dependent on their husband, and four were in a polygamous marriage. Women’s decisions were based on realistic assessments of the options available to them in a context where economic survival and keeping their family intact were priorities. Dissolving the marriage could disrupt social ties, result in women’s destitution, loss of their children and bring shame to their family.

*… I’m a woman who protects my home and my husband and my family, my kids … I want someone to advise me to do something that benefits me, but without wrecking my relationship with my husband. Why? Because there are children in the middle. I don’t want a wrecked home … Why should I scatter myself, get a divorce and all that. I don’t want any of that to happen. All I want is to live, like others are living. I have a desire to live for the future*. [SW03, Woman, 27 years]

Providers referred to a culture of silencing and normalising of violence where women were customarily blamed for their husband’s abuse. Some providers did not want to push women (or themselves) too far with their questioning, instead preferring to facilitate a staged disclosure (*I would try to pull the thread*) where trust and boundaries could be negotiated over time. Their narratives illustrate the ways in which they regulated their behaviours to avoid negative consequences, although in doing so they were unintentionally policing the boundaries of women’s behaviour and preserving the status quo.

*I knew their families are really difficult and would not understand. She might slip one day and say that she went to this doctor and he asked me 1, 2, 3. There’s a great chance they wouldn’t understand, so I was very superficial. Very superficial. I didn’t go deeper*. [HP02, Doctor, Male]

*Back when we were dealing with the [high risk] case [she] told me that she wouldn’t notify me about any more risky situations which could potentially endanger our lives. Even though police officers might be able to protect us at our workplace, they wouldn’t be able to ensure our safety in the privacy of our homes or on the streets … Never consume yourself in critical cases and always know your limits* [HP03, GBV Focal Point, Female]

Provider responses were, at times, entangled with a felt obligation to maintain the reputation of the women and their families.

*We were reluctant to go after the young woman to ask her to take a pregnancy test for fear of getting into trouble. Hence eventually we made our minds to rule out that option. That was one of the cases I dealt with and discussed with the training team. We debated how we should have acted during that time, what we should have done if the pregnancy test had come back positive … I would have reported the case to the Case Manager*. [HP05, Nurse, Female]

Women’s experiences with health care providers in other healthcare settings revealed how embedded these attitudes were. A woman [SW02] described being transferred to a hospital for a fractured ankle after being attacked by her husband. Although she identified her husband as the abuser, the incident was documented as “HBO” (hit by others) in her medical record by the attending doctor who told her: *I didn’t see who hit you. You’re saying you’ve been hit by your husband, but I can’t write that. You can report to the police that you’ve been hit by your husband*. Another woman [SW03] who required treatment for head injuries reported being advised by the doctor to be cautious because her husband could be arrested (*he even admitted that he and his wife got into an argument and he got upset and hit her).*

Some encounters revealed how strongly interactions were shaped by the normative context. Advice giving reinforced the notion that women should try to understand the source of their husband’s abuse and keep a peaceful home to avoid conflicts.

*The husband is pressured by those around him, his family, or psychologically he is … financially … So you have to diagnose the cause if it’s because of those around him or is financial. If financial, you have to tell the abused woman to have patience on her husband, be more patient … You have to befriend her and tell her how to get close to him in order to win him over …* [HP02, Doctor, Male]

*P: … We advised her to solve the dispute with him prudently. We advocated for the use of mutual understanding and common sense, in spite of the difficulties.*

*I: … what do you plan to tell her when she comes for follow-up?*

*P: We plan to give her guidance on how to deal with her husband, how to avoid getting hurt by him and where to go if her problems get out of hand.* [HP04, Head of Clinic, Nurse, Female]

A doctor [HP01] reported mediating in a domestic violence case by offering to speak to the woman’s husband with her permission. His esteemed position within the community as an older, male doctor, engendered feelings of trust among his patients who would seek him out for advice in an almost paternalistic way (… *the people and patients consider me as a father or as a big brother for them. They listen to what I say …*). The doctor perceived the cause of the violence to be disagreements over how money should be spent, as opposed to the woman’s lack of decision-making power within the marriage and counselled the husband on a few occasions who ceased his use of violence.

*So, look we solved things, he used to always hit her and even one time she said he tied her up with rope. He came and told me straight up that he treated his wife this way and that way, and [he] used to hit …*. *So, after getting closer they went back and now things are going well and I followed up with them. We hope everyone will be like that, to respond and get things back to normal.* [HP01, Doctor, Male]

Families were also complicit in governing behaviour through monitoring women’s movements which limited their access to support through HERA *(… usually my husband sends people after to me to watch me*).

*It’s been about two months that I don’t go out at all … I’ve only come here to the clinic. I don’t leave the house, I don’t visit my parent’s home. I’m even forbidden from visiting his relatives* [SW03, Woman, 27 years]

One of the GBV focal points reported that the abuser in one case that she was involved with was paid by family to remain silent about his daughter-in-law’s pregnancy, a consequence of his sexual violence toward her. The woman’s mother-in-law who was aware of the abuse was reported to have physically hurt her in order to procure a miscarriage. The strong influence of clan-based social relationships was evident even when women sought help from the police, whose efforts were ineffectual under tribal resolution where mediation was customary.

*They sent family men to talk to me, to convince me [to return] because the next week was school. Who is going to take care of them? Who will be with them?... But the men insisted … and I went back … Also there was some kind of pull with the police [woman reported her husband to the police]. Someone from [name of family] works in the police … but he made everything go smoothly with the police and that’s it, when he makes a call to the police they keep quiet. After a week or two nothing happened. The police didn’t do anything.* [SW02, Woman, 30 years]

#### Confidentiality and controls over knowledge

The safe and proper functioning of the care pathway was dependent upon the extent to which knowledge about domestic violence disclosure and support offered could be controlled. Plans to conduct community awareness raising sessions were abandoned on the advice of some stakeholders. It was felt that publicising the role of primary health care clinics in the management of domestic violence in the community would create paradoxical effects, as it was likely to result women being prevented from attending the clinics. Consequently, they conducted awareness raising sessions in the pilot clinics with female patients.

Clinic case managers and women resorted to subterfuge to create opportunities for follow-up (*We make cover stories … we tell her you have an appointment for your child at this day, but we actually want to see her*). Some women were referred to the clinic case manager under the guise of taking vital signs thereby creating opportunities for them to *win the woman’s trust and have a conversation with her*.

*P: The woman … was escorted to the clinic by her father-in-law on her second appointment. He stood guard at the door of the clinic and kept asking why it took her so long to finish. The moral of the story is that battered women don’t have the luxury to leave the house and move around freely.*

*I: How did you respond to the father-in-law in that case?*

*P: We told him that [she was] was filling a medical report and that the delay was due to computers being down. Where in reality, we were trying to hold her off until the psychologist got to the clinic.* [HP04, Head Nurse, Female]

*I: And you’re allowed to come here, to the clinic?*

*P: Not always, but I always try to come. Even if I have to lie for my benefit or the benefit of others, that’s okay.* [SW04, Woman, 29 years]

The clinic was a safe space where women negotiated their needs and what information they wished to share. The Ministry of Health required that all cases of domestic violence be entered into the confidential clinic register. However, there was no written protocol and providers engaged in a number of informal practices to accommodate women’s preferences as some were reluctant to have anything written down for fear of being discovered. Providers exercised discretion regarding documentation in order to maintain the trust of women and protect themselves from retaliation. Such practices may also be thought of as governing behaviours.

*… one woman she completed the [domestic violence] questionnaire with everything fine [*i.e. *no disclosure of violence] and then she said “I want to tell” and she started talking … There are these cases where woman do not want people to know about her being tortured at home.* [HP06, Family Planning Nurse, Female]

*P: The doctor asked that of me [to document the domestic violence].*

*I: And what did you say?*

*P: He told me “whatever you want to agree” because I refused.*

*I: Can you tell me why you refused?*

*P: Because I wanted the whole subject to be private.*

*I: Okay so you feel as soon as it gets written down, it isn’t private anymore?*

*P: Yes, because I worry that it might get to someone.* [SW04, Woman, 29 years]

Documentation remained a contentious issue, linked to professional accountability, a need for tangible evidence of the abuse and a lack of clarity about whose role it was to document.

*One time she was beaten by her husband so hard, she came to the clinic complaining of pain in the stomach. I examined her and figured out it was abuse and immediately referred her to the hospital. But I am not very sure if this case was documented … She was documented as a pregnancy follow-up, but I’m not sure if she was documented as a case of abuse. If I see signs of abuse on the face or body of the victim, or if she reaches out to me and tells me that she is abused, as a doctor I write what I see. For example, if a patient claimed she was beaten, but I couldn’t see any signs relating to her claim, I document the patient was beaten as she claims. So, I use the word ‘claim’ which I wouldn’t use if I witness evidence of abuse. We only document those who admit that they were abused. I can’t document a case if the victim denies being abused, even if I saw signs of abuse*. [HP10, Gynaecologist, Female]

Clinic case registers indicate that a higher number of domestic violence cases were documented at Clinic 1 in Area C compared to Clinic 2 in Area B (15 versus 6 cases). Clinic 2 was in a small town, which may have raised concern among women about boundaries in a community where there was more familiarity between patients and providers. However, there were conflicting views about this, as one woman [SW02] from Clinic 2 felt reassured by the familiarity between her and the nurses (*they know me, they’ve gotten used to seeing me because we’re all from the same town. I know and was reassured that she wasn’t going to talk about it to anyone. That she wouldn’t spread talk around*). Conversely, a gynaecologist at the same clinic [HP10] felt that women patients trusted her because she did not live in the community. She reported that a pregnant woman refused to talk to the clinic case manager who lived locally. A major shift in practice involved the use of private space and greater awareness that trust between women and providers was not implicit (*… we promised them it would stay between us and I would try to help that it doesn’t go outside of the family*)*.* Clinic 1 created a dedicated counselling room although prior to HERA women discussed abuse in front of other providers who shared consulting rooms.

The porosity of the boundary between clinic and community was substantiated in one case where a male doctor’s questioning about domestic violence was misinterpreted by a female patient as harassment, who then informed her husband. The incident was shared with men in the community and the identity of the doctor was exposed. A nurse at the clinic reported that her husband was made aware of threats to hurt the doctor (*they hammered my husband with questions about the doctor’s background, morals*). The incident was escalated to the Directorate GBV focal point and the doctor was advised to inform the clinic case manager of any future patients he suspected were experiencing domestic violence, rather than make inquiries himself. Two of the three male health care providers interviewed reported using a similar approach with female colleagues to avoid misperceptions of cultural inappropriateness.

*Some [women] would come to the clinic agitated or acting weird, which would raise my suspicion that something wrong was happening. As a male doctor, I couldn’t reach out to them and offer them help directly. Therefore, I used to call the head of the nurses and tell her to speak to this patient in private. In fact, this happened more than once. And you know it is easier for women to open up and speak to another woman, especially in our culture*. [HP11, Director of the Clinic, General Practitioner, Male]

In a very severe case of violence, knowledge was carefully controlled to protect the woman, who was suicidal, and those involved in her care. After consultations between the GBV focal point, Health Directorate and the Public Prosecutor, the woman was taken by ambulance to a large government hospital in an area under the Palestinian Authority (contravening the abuser’s wishes to have her transferred to a small hospital). The physician at the clinic who issued the medical report was not informed of the reason for the transfer in order to protect him from family retaliation. Medical documentation of the woman’s injuries was undertaken at the hospital and used to arrest the abuser. Responsibility for the case was diffused over a network of professionals and institutions thus reducing the risk of retaliation (*blame for unveiling the incident would fall on the institution as a whole, and no single person would be deemed liable for exposing the truth of what happened*).

### Relational restructuring

#### Improvisation of practice and transformation of roles

An emergent feature of the intervention was the ability of providers to improvise the work and adjust their accountabilities to each other in response to contextual barriers. The Ministry of Health supported the creation of the clinic case manager role within HERA, assumed by female nurses, and which helped to bridge a structural gap that previously existed between the clinics and the Directorate level GBV focal points. This was due to a lack of clarity about whether the role of the GBV focal point was purely administrative or provision of support to women. In HERA, their function was more clearly articulated as offering guidance to the clinic case managers when dealing with complex cases and coordinating further support for women outside of the clinic. The involvement of the GBV focal points and clinic case managers in the training helped to reinforce the function of these roles to providers. The relational restructuring enabled the diffusion of responsibility throughout the health system, which increased providers’ comfort in identifying domestic violence.

*I: What about other members of staff? Have the roles become clear to them as well?*

*P: Yes, they have. The training has opened the door for cooperation between the team members. Since the HERA training, we’ve dealt with many domestic violence cases referred to us by doctors at the clinic.* [HP04, Head Nurse, Female]

*… These days we actually even taught the reception staff, the pharmacists and other health care providers in this clinic of how to identify a potential violence victim and to directly refer the case to us. So now it has become a cooperative work*. [HP09, Clinic Case Manager, Female]

However, referral of women from the clinic to the GBV focal points did not occur as anticipated as few accepted external referrals. Some women were restricted from travelling unaccompanied and there was immense stigma attached to seeing a psychologist (*They get scared when they hear the word psychologist*). Furthermore, women wished to keep their family intact and felt that external referral could compromise this. The workability of the care pathway required modification to designated roles. Subsequently, cases of domestic violence were maintained in the clinic where women’s encounters with the clinic case managers became the focus for therapeutic intervention. Their role in providing a first line response and onward referral was transformed into one that involved alleviating women’s psychological distress. The clinic became a space for respite where women could *vent* their frustrations and have someone (safely) bear witness to the violence they were experiencing (*I was looking for people to hear me*). Women described feeling comforted, but conceded there was little that the case managers could do beyond providing emotional support and safety advice if they chose not to be referred. Clinic case registers show that only 5 out of 18 documented cases accepted an external referral (i.e. outside the clinic).

*P: Before I talked to her, I felt strangled. I felt strangled like something was holding me around my neck. But after a little I started talking to her I was getting worked up. I wanted to cry and scream, and scream and scream [voice gets higher]. It’s not that I needed to talk, I needed to scream*.

*I: How do you feel now after you’ve spoke to … the nurses?*

*P: I feel like I’ve released a bit … my mental state is relieved.* [SW02, Woman, 30 years]

*… a pregnant woman with a broken leg due to abuse approached me in the clinic. I offered her referral, but she refused because she didn’t want to lose her kids. Most of the women in this society come to me just to speak out and just to feel like someone listens to them. So, we actually reach a dead end in the management process. The solution in my opinion is creating a national system to support abused women along with their children … and I repeat along with their children* [HP10, Gynaecologist, Female]

### Synergies with capacity and potential

#### Limited role legitimacy

Whilst the resourcefulness of the clinic case managers resulted in a modified care pathway, it was an unanticipated outcome which impacted the sustainability of HERA. They did not feel adequately prepared for their evolving role in dealing with the psychological effects of abuse and felt that their efforts to improvise could do more harm than good. Furthermore, dealing with domestic violence cases was only part of their remit as they had oversight for other health programmes. Burgeoning workloads and lack of staff led to de-prioritisation of domestic violence, which was perceived to have lower levels of accountability to the Ministry of Health compared to other work. In order to manage these deficits providers mobilised a tacit rule about other work taking precedence, which may have reflected the position of some managers within the Directorate (*The case manager doesn’t afford the time to work full-time on the programme … She’s compelled to prioritise her nursing work, which she’s accountable to do over domestic violence care. We hope to solve this issue with decision makers in the future*).

The limited decision-making power of Directorate Managers to re-direct resources to the clinic case manager role (e.g. training, clinical supervision, case management and re-allocation of workloads) further undermined their role legitimacy. There was a reported decrease in the number of domestic violence cases identified due to the heavy workload of the case manager (*they were occupied with their original work … There was no specific personnel dedicated for recognising and following up domestic violence cases*). Providers in both clinics described similar work pressures and some were of the view that a psychologist based in the clinic would better serve women’s needs. The individual and collective commitment of providers began to ebb away in the course of the pilot under the burden of competing priorities.

*It’s imperative that we have to cope with increased workloads due to the nature of our job … I have to serve large numbers of patients on a daily basis … The drop in the number of documented cases was attributed to the heavy workloads in the past month. I bet that if we had fewer workloads or experienced people dedicated to handling domestic violence cases, the outcome would be more efficient than its current state. There would be more room for guidance and counselling, activities and lectures … If we hadn’t been swamped with nursing work all the time, we would have divided the duties between the team members and assumed a more active role in combating violence.* [HP04, Head Nurse, Female]

#### Lack of “higher-level” commitment affecting collective motivation

There was an expressed need for *higher-level* organisational commitment and the lack of congruence in positive values between the clinical team and Directorate managers negatively affected providers’ motivation. Not all Directorate managers and supervisors were invited to training which may, in part, explain these perceptions.

*We wanted them to be closer. If there was more interaction with management it would have been better … we were even more enthusiastic than the health directorate itself, than the supervisor, the minister, the manager, and so, it would give me some motivation. But they tell you, “do as you please, if you want to deal with them [domestic violence cases] welcome, if you don’t want to, as you like” … If we have support from the governorate, the Ministry of Health or the associated authorities* … [HP01, Doctor, Male].

Issues affecting capacity and potential were also highlighted in relation to the training as evidenced in the attrition rates at the three reinforcement sessions (73%, 55% and 51% respectively). Providers spoke favourably about the training and reported increased comfort in asking about domestic violence and being more attuned to risk markers. Both trainers reported that providers valued experiential learning and case discussion. Yet it was challenging to sustain the positive effects of training as some of the providers rotated to other clinics, which weakened the collective work that had developed. Additionally, new staff rotating to the clinics for brief periods were not as invested in training as reported by one of the trainers (*In some areas the staff said it’s not part of our role, why should we stay in the session? The core staff, the nurses and doctors stayed in the session the whole time*). Furthermore, staff cover was not arranged for providers who attended training resulting in disruptions because attending to patients was prioritised over training. The trainers recommended conducting the training outside of the clinic, potentially in the Directorate offices, which would also help to foster higher level commitment.

*Some of them used to come in and go out and see some patients and come in again … the trainees were not fully free for the training, some of them* … *I mean imagine that you are giving a training and every few minutes you are being interrupted by people coming in and people going out... It was not easy.* [Trainer 2]

*The clinic is a vital primary healthcare unit with a remarkable number of patients attending its outpatient clinics on a daily basis … It was impossible for staff to attend the full sessions because some needed to fulfil duties during the working hours*. [HP03, GBV Focal Point, Female]

## Discussion

**Fig. 4:**
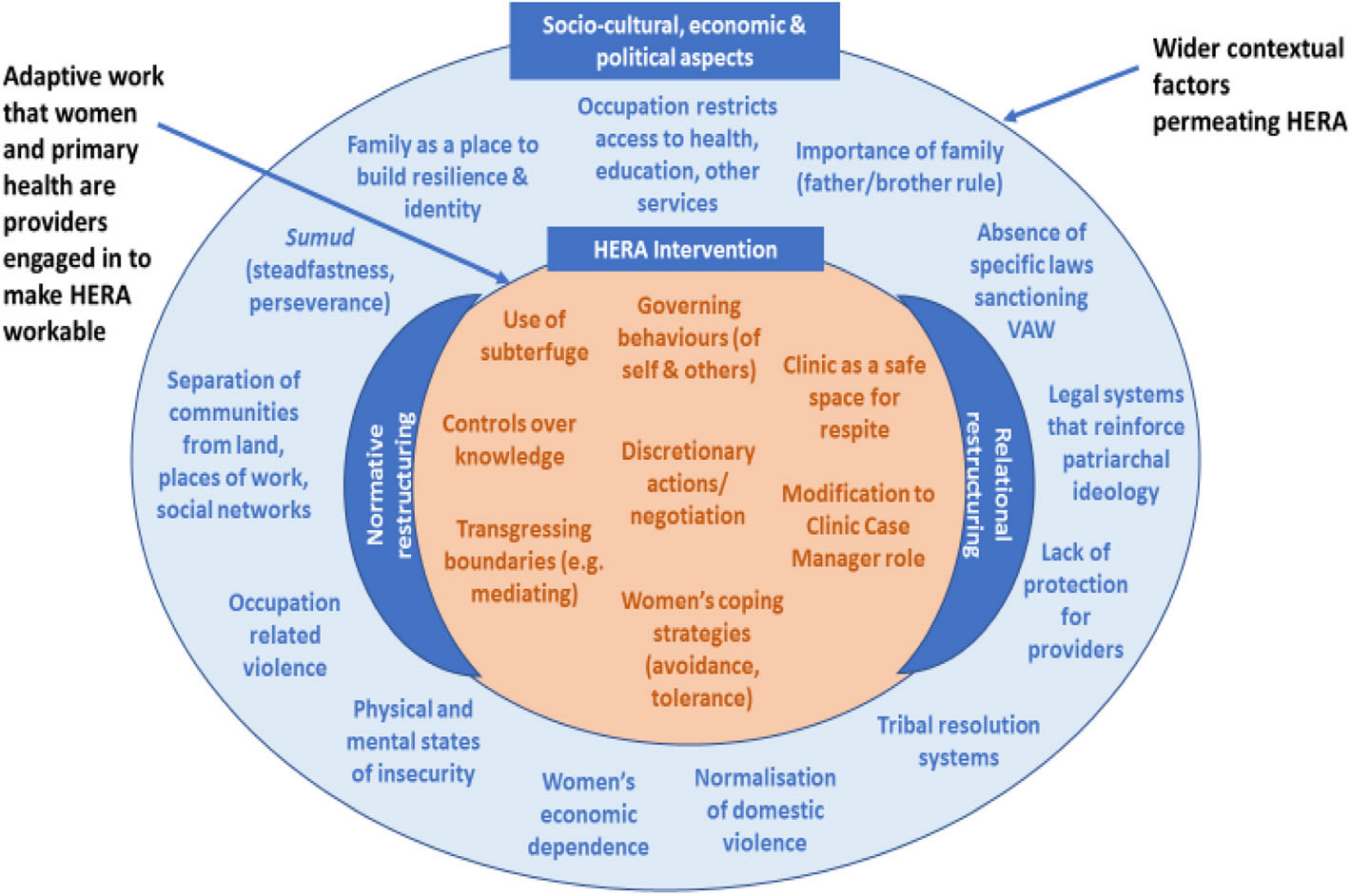
Adaptive work in a dynamic context

To the authors knowledge, this study is the first use of ENPT in the evaluation of a health system intervention to address domestic violence in a low-and-middle-income country. A systematic review of feasibility studies and process evaluations of complex healthcare interventions that used NPT or ENPT included five studies from low-and-middle-income countries (all using NPT), although none integrated responses to violence against women [38]. The study highlights the importance of considering context in the design, implementation and evaluation. We demonstrate how intervention-context interactions affect fidelity to the HERA model in Palestine and how, through adaptive work, participants negotiate multiple and interlinked contextual constraints (Fig. 4).

The political occupation restricts women’s movement and access to support services, whilst the concomitant lack of security and police protection leave providers and women feeling exposed to acts of family retaliation. This is interwoven with the strong influence of cultural values that also affect women’s choices because of their primary role as nurturers within the family. Women’s responses to these barriers illustrate how closely their needs and interests are bound up with those of their family. They utilise the clinic as a safe space in which to diffuse emotional pain and frustration, exhibiting passive (e.g. avoidance and tolerance to maintain harmony) and active (e.g. mobilising support through the case managers) coping mechanisms. Their decision not to use referral services may be understood as a hidden form of agency as they struggle for physical and economic survival in a highly constrained context, where political and legal systems fail to protect their rights and safety. The concept of *Sumud* is also relevant here and deeply embedded within Palestinian culture. It relates to ideas about personal and collective steadfastness in the face of daily challenges, but it is also a socio-political concept which refers to ways of surviving in the context of occupation, poor infrastructure and lack of resources [39, 40]. *Sumud* can be used at the individual, family and community level and relates to tangible resources that support basic needs and intangible resources such as belief systems, religion and social and family support [41]. Makkawi [42] states that girls are more likely to develop their sources of resilience inside the home, as this is believed to be the most secure environment for them due to the lack of security under the occupation. All but one of the women in our study were in a polygamous marriage and all of them were economically dependent on their husband or family. Their decision not to access external referrals and keep their marriage intact can be understood as a strategic one to avoid the loss of economic and social support or their place in the community, but also an act of resilience in the context of limited alternative options. Interviews with women during the formative phase of our study supports the finding that remaining in the home is often a tactical decision and motivated by the desire for financial protection [43].

In Palestine, the family is an important structure for building individual and collective resilience against the occupation, but this sits uneasily alongside other forms of oppression, such as domestic violence. It creates a paradox for women because their identity as women subjected to gender inequalities cannot be divorced from their identity as Palestinians struggling under the occupation. Women’s attempts to preserve the coherence of family as a source of resilience must co-exist alongside inequality and conflict within the family. As reported elsewhere [21], our findings highlight how women see the family as a means of achieving certain interests (i.e. survival for themselves and their children) even though other interests (i.e. gendered relations) are undermined. There is also a degree of resonance with Kandiyoti’s [44] work on “*patriarchal bargains*” in male dominated systems in the Middle East. Women negotiate, accommodate and strategise within a set of constraints in order to maximise their security with varying potential for active or passive resistance in the context of oppression. Patriarchal bargaining shapes women’s rational choices, but also influences the unconscious aspects of their gendered subjectivity. Researchers conceptualising the agency of women in violent relationships caution against overuse of the “victim-agent binary” to understand the experiences of marginalised women in less developed settings. The narrow focus on observable actions such as documentation and use of services tends to obscure the myriad ways in which women counteract the violence whilst remaining in the relationship [45]. It is challenging to measure the success of interventions using such metrics because studies show that a minority of women (6%) in low-and-middle income countries who experience domestic violence use referral services [46, 47]. In Palestine, whilst 40% of women are aware of support organisations, only 1% seek psychosocial or legal assistance or police protection [3]. Future research should aim to capture women’s survival strategies and their effectiveness in preventing further harm [48].

Health care providers work within normative structures which also influence their actions. The findings draw attention to the precarious position of providers as they negotiate fears about retaliation for interfering in what is perceived to be a private family matter, and meeting the needs of women patients for whom referral options may be severely limited. Whilst they take care not to fracture social relationships and compromise women’s economic survival, their actions (or lack of) inadvertently reinforce cultural acceptance and normalisation of violence against women. Studies of Palestinian providers demonstrate their views may align with gender norms that position women as subordinate to men within the family and misconceptions about domestic violence. This includes a tendency to attribute wife abuse to characteristics of the woman or perceived transgressions of her role [49, 50]. Nonetheless, whilst possibilities for restructuring are limited, certain features of HERA are malleable as participants mould them to fit the context [6]. In spite of contextual constraints providers and women engage in adaptive work (subterfuge, active and passive coping, negotiation, controls over knowledge, governing behaviours, improvisation of roles) to engage safely with HERA.

The transformation of the clinic case manager role is a significant emergent feature of HERA, revealing how providers continue to invest in overcoming shocks and turbulence within the context. The collaborative nature of the HERA referral pathway and clear roles appear to enhance providers’ readiness to address domestic violence. However, the clinic case manager role will be difficult to sustain as they are not trained to provide ongoing psychological support to women beyond a first line response [32]. Since few women want to access support from external services, the onward referral of women from the clinic to the Directorate based GBV focal points (external to the clinics) is unlikely to occur as predicted. Furthermore, there is an absence of standard operating procedures or policies that provide legitimacy for the clinic case manager role as domestic violence work has to compete alongside other roles they are tasked with. This may, in part, explain providers’ declining commitment to HERA, both in terms of attrition at the reinforcement training sessions and their perceptions of domestic violence as having a lower level of accountability compared to other work. These findings highlight the synergistic links between capability (experienced workability and integration of the intervention in context), capacity (social-structural resources devoted to the intervention and institutionally sanctioned rules that give structure and meaning to roles and relationships, which in turn define how people behave) and potential (individual and collective readiness). A recent qualitative meta-synthesis highlights the link between health practitioners’ readiness to address domestic violence and the importance of creating an *authorising organisational environment* (e.g. through policies and procedures). Collective care strategies that involve specialists outside the health care setting and allowing time to do the sensitive work also facilitate practitioners’ readiness to engage in domestic violence work [51].

Findings from the formative phase of our study in Palestine draw attention to the partial readiness of the health system to integrate a domestic violence response. Some issues were addressed prior to intervention development (e.g. clear roles in the referral pathway, the nomination of clinic case managers, the involvement of GBV focal points in the training, improving privacy and confidentiality, raising community awareness, and addressing safety issues during training) although some preparedness gaps persisted. In particular, in relation to the lack of financial resources, incongruent values (tension between patriarchal views on domestic violence and gender equitable norms) and unclear and unsupportive leadership structure at district level which weakened the agency of GBV focal points to manage and refer women [5]. The de-prioritisation of domestic violence response due to capacity issues, the lack of high-level health system support and weak policy implementation are also indicated in the evaluation of primary health care interventions to strengthen domestic violence response in New Zealand [52] and Brazil [53, 54].

Findings from the evaluation of HERA highlight how factors that potentially undermine the intervention may also support it. Permeable boundaries between the clinic and community can facilitate disclosure of domestic violence when there are relationships built on trust and familiarity, yet they pose a risk when information is inadvertently conveyed to the wrong people. The discretionary actions of providers and negotiations with women (e.g. regarding documentation) enhance trust, which help to engage women in HERA and referral to the case manager. However, the absence of documentation can be detrimental to women who require evidence of domestic violence for legal reasons. Additionally, since clinic data are used by the Ministry of Health for epidemiological surveillance, undocumented cases result in undercounting and undermine efforts to advocate for the critical role of health care providers in a societal response to domestic violence. Mediation and advice that focus on harm minimisation (i.e. avoiding and removing stressors, modifying and pacifying behaviours) may well keep women safe from escalating violence in a context where few viable options exist [48]. However, when providers advocate the use of these strategies, there is potential to reinforce perceptions about women’s culpability in abusive relationships, unless they are supplemented with strong messages that women are not to blame and that referrals options are available. The paradoxes that providers encounter should inform further development of the HERA training programme in Palestine. Protected time for individual and group support where sensitive and challenging cases can be discussed are essential to reinforcing provider readiness.

### Strengths and limitations

Flexible use of ENPT enhanced the analytical process by encouraging new ways of thinking about the data, and helped to surface how participants negotiated contextual constraints. Although some users of NPT or ENPT have critiqued the vocabulary for being difficult to understand and having overlapping constructs, we did not encounter these problems. We chose not to employ ENPT as a framework with which to code qualitative data, were selective about which constructs to focus on and did not use ENPT language in the interview guides. We were able to elicit rich accounts from providers, case managers, GBV focal points and women. However, the sample size was small, particularly with regards to women participants. Despite reassurances of confidentiality, local researchers experienced difficulties in recruiting women from the clinics. Clinic case managers were responsible for introducing the study to women, but reported that many women were concerned about potential exposure. It was also felt that the particular family background of some of the women might have exposed the researchers to potential threats if women’s participation in the study became known in the community. The inclusion of more women and those in paid employment may have yielded a wider range of experiences, particularly with respect to the choices available to them, offers of referral services and their willingness to access them. Furthermore, the inclusion of other stakeholders including funders of health services and other Directorate managers would have provided a richer account of how different groups made sense of the intervention and the ways in which this influenced collective action. It would also have helped to understand the influence of higher-level managerial support within the health system on issues at the clinic and provider level. Finally, it is likely that the evaluation only began to capture implementation processes in the seven-month evaluation period. May et al. [6] advocate the use of longitudinal accounts that use mixed methods to understand how practices become routine (embedded) and sustained (integrated) over time and between settings.

## Conclusions

We highlight the importance of considering context in the design, implementation and evaluation of health system responses to violence against women. The use of ENPT helped to surface practices that providers and women engaged in to make HERA workable (and safe) as they negotiated multiple contextual constraints. These practices reflect a set of ‘real world’ activities were not part of the intended HERA model. It is the adaptive work that made the care pathway possible and enabled providers and women to deal with the perceived risks. The findings have implications for the transferability of evidence-based interventions on health system responses to violence against women in different contexts. Although the findings are limited by the small sample size and the duration of the evaluation, they have important implications for how HERA can be resourced to ensure its sustainability. Despite strong political will, providers touched upon deeper issues related to the lack of tangible higher-level support which has been reported in other studies [52, 53, 55]. The subsequent de-prioritisation of domestic violence and the limited role legitimacy of case managers are inherently linked to insufficient capacity and providers gradually withdrawing their commitments. There is a need to explore how psychosocial support can be offered to women within primary health care clinics, and how to legitimate roles in standard operating procedures. Engaging Directorate level supervisors and managers within domestic violence training programmes, including awareness of their leadership role in supporting providers and enabling change can help to build a more cohesive response throughout the health system.

## Data Availability

The data generated during the current study are not publicly available due to the highly sensitive nature of the topic under investigation. We will assess data for release at the close of the study. Data with low sensitivity will be released openly at the end of the stated embargo period. Data which retains a small risk of re-identification, even after use of pseudonyms and removal of direct identifiers, will be shared as controlled data. Requests for access to this data will be assessed by the University of Bristol Data Access Committee. Data which poses a high risk of participant re-identification and cannot be de-sensitised will not be shared.

## Abbreviations

ENPT: Extended Normalisation Process Theory
GBV Focal Point: Gender Based Violence Focal Point
HERA: Healthcare Responding to Violence and Abuse
NPT: Normalisation Process Theory
